# High-Standard Non-Surgical Endodontic Treatment and Outcome: A Retrospective Follow-Up Study on Self-Assessment of Grading and Case Difficulty in an Academic Setting

**DOI:** 10.3390/dj13120571

**Published:** 2025-12-02

**Authors:** Ahmad Naweed Faizi, Inge Fristad, Sivakami Rethnam Haug

**Affiliations:** Department of Clinical Dentistry, Faculty of Medicine, University of Bergen, 5009 Bergen, Norway; a.naweedfaizi@gmail.com (A.N.F.); sivakami.haug@uib.no (S.R.H.)

**Keywords:** standard of excellence, success rate, tooth survival, quality evaluation, self-reflection, case difficulty assessment

## Abstract

**Background/Objectives:** Endodontic treatment quality can be graded from A to D according to international standards. This study aimed to evaluate radiographic treatment success of high-standard endodontic treatments (Grade A). A secondary aim was to assess the students’ self-perceived assessment of case difficulty. **Methods:** A retrospective analysis was conducted on root canal treatments (RCTs) performed by undergraduate students at the University of Bergen between January 2012 and December 2014. Data related to endodontic treatments graded as A, along with a postoperative self-perceived assessment of case difficulty, were extracted from patient records. Statistical analysis was performed with a significance level set at *p* ≤ 0.05. **Results:** Out of 1149 RCTs, 462 (40.2%) were classified as Grade A. Among these, 350 teeth had at least one recall after one year. Out of the 350 teeth, 312 were successful (89.2%), 6 (1.7%) were failures and 32 (9.1%) were extracted due to factors unrelated to endodontic infection. After exclusion or inclusion of extracted teeth, the success rate was 98.1% and tooth survival 90.9%. Patients under 56 years of age, and teeth with indirect coronal restorations, had significantly better tooth survival. Students significantly underestimated case difficulty (*p* < 0.01). **Conclusions:** High-standard endodontic treatments can achieve a high success rate. Self-assessment of endodontic treatment and accurate assessment of case difficulty using relevant tools serves as an important educational aid, potentially contributing to enhanced knowledge and improved clinical decision-making.

## 1. Introduction

International standards have suggested a quality classification system into four categories [1,2], and, according to the Norwegian Ministry of Health, a quality evaluation system for continuous learning and improvement is mandatory in the health care service [3,4]. In the publication “Self Assessment Manual and Standards for health services” (SAMS), the Faculty of General Dental Practitioners at the Royal College of Surgeons of England, in line with the Californian Dental Association, defined four quality standards in general dental practice where clinical patient outcome can be measured against generally accepted norms [1,2]. For evaluation of endodontic treatment, we have translated and adopted these standards into our quality evaluation system. These standards, graded as A, B, C and D, are concerned with clinical outcomes rather than techniques, and are intended to be educational [2] (Table 1). Standards A and B indicate highly satisfactory or satisfactory outcomes, respectively, while standards C and D are unsatisfactory and indicate that the patient may suffer or has suffered damage to the teeth and/or oral tissues during treatment. Self-assessment requires a degree of discipline, both to initiate the process and to remain honest with oneself. The function of the exercise is to examine one’s own standards and to decide, in the opinion of each dentist, what the appropriate standard should be. The purpose of incorporating self-assessment in an academic environment is to instill continuous self-evaluation and commitment to high-quality treatment among practitioners.

Endodontic treatment aims to prevent or treat apical periodontitis, following strict aseptic treatment protocols [5]. The presence of adverse events, otherwise known as mishaps, procedural or iatrogenic errors, are commonly encountered during endodontic treatment and may negatively affect the prognosis [6]. These events can cause anywhere from potential damage, reversible damage or irreversible damage to the patient and result in immediate or future adverse outcomes. Legal allegations during or after endodontic treatment are considered an increasing problem [7,8].

Grade A is a standard of excellence where treatment is performed without any adverse events or deviations from the ideal result, thus expected to have the highest prognostic potential among RCTs. However, a distinction should be made between standard and treatment outcome. Whereas SAMS are concerned with the technical standard of the treatment, evaluated based on clinical performance and immediate postoperative radiographs, the success or long-term outcome of the treatment is determined by radiographic follow-up examinations of the periapical status [9]. Our previous study on cases graded as D showed that 46% of the teeth were still present without periapical pathology after a 4- to 5-year observation period [10]. However, 66% of these teeth had received surgical interventions, including root resections or surgical retreatments.

In clinical dental education, the students should follow accepted treatment protocols (Supplementary Materials), be able to perform self-assessment and self-reflection, and finally justify and discuss treatment plans and results. By using the SAMS to evaluate treatment performance, dental students develop essential skills in critical assessment and self-reflection, emphasizing the importance of the ‘judgment factor’ in becoming proficient future dental practitioners [1,2].

This is, to the best of our knowledge, the first study that relates high-quality endodontic treatment and outcome based on a grading system intended for quality control in the health care system. In epidemiological studies and most follow-up studies, the overall prognosis does normally not discriminate between aspects connected to quality, which is expected to have a considerable impact on the prognosis according to the definition and intention behind the international standards [1,2]. The aim of this study was therefore to examine the effect of high quality on the outcome of orthograde endodontic treatment. Further, the student’s subjective postoperative perception of case difficulty for cases graded as A was evaluated.

## 2. Methods

In this retrospective study, with a follow-up period of up to 11 years, we included RCTs performed by undergraduate 4th- and 5th-year students, between January 2012 and December 2014 at the Department of Clinical Dentistry, University of Bergen, Norway. A data search was performed in the electronic patient journal system (Version 7.1.163; Opus Systemer AS, Planmeca Group, Helsinki, Finland).

All students registered occurrences of adverse event(s) and self-assessed their treatment performance according to the SAMS into one of four grades (Table 1), with treatments of high quality, Grade A, being the focus group in this study.

Grading of the treatment quality was reviewed by a clinical instructor (specialist in endodontics), who either approved or contested the appropriate grade in the patient records. A list of registered adverse events used in the evaluation is included in Table 2. Additionally, the students predicted the prognosis and subjectively self-assessed case difficulty postoperatively. To evaluate the accuracy of the student’s subjective ability to evaluate case difficulty, an independent objective case difficulty assessment of all cases was performed by one of the authors (SRH), using the American Association of Endodontists (AAE) case difficulty assessment form [11].

### 2.1. Inclusion and Exclusion Criteria

Completed RCTs, including primary treatments and non-surgical retreatments classified as Grade A were included in the study. All patients were offered a one-year follow-up, but unattended patients were included if radiographs were otherwise available during the follow-up period. Teeth missing recall data were excluded.

### 2.2. Evaluation of Treatment Outcome

The treatment results were graded as success, uncertain or failure based on criteria by Halse and Molven [9]. Briefly, success was defined as no radiographic periapical radiolucency with normal width of the periodontal space; uncertain as reduced radiolucency or widened periodontal space; and failure as pathological finding with increased or unchanged periapical or juxta-positioned radiolucency. After calibration, using digital radiographs of 40 root-filled teeth to arrive at uniform interpretation and application of the criteria [9], the follow-up radiographs were examined by two independent observers (ANF and SRH). In case of disagreement, a third examiner was included (IF) before a joint decision was taken. For dichotomization, asymptomatic cases, not yet completely healed, were recorded as successful.

In addition to evaluating endodontic success and failure, survival of root-filled teeth was also recorded. In this context, tooth survival refers to teeth retained or still present during follow-up, independent of radiographic success or failure.

### 2.3. Ethical Considerations

The Norwegian Ministry of Health and Care Services’ guide to the Health Research Act, defines quality assurance as projects, examinations and evaluations that aim to check that diagnostics and treatment in fact gives the intended results. The need to consent to participate was waivered by the Institutional Review Board and the Regional Committees for Medical and Health Research Ethics West, Norway, as this project was categorized as a quality assurance study (REK 813936). The study was approved by the Department of Clinical Dentistry. Throughout this process, all regulations for GDPR, as legally mandated by the EU for all EU and EEA countries, were strictly adhered to.

### 2.4. Statistical Analysis

Statistical analysis was performed in STATA 18 (StataCorp LLC, College Station, TX, USA) and Statistical Package for Social Sciences (SPSS, Statistics for Windows, version 29.0 released 2023; IBM Corp., Armonk, NY, USA). The mean value of the study cohort, 56 years, was used to dichotomize the data to study the effect of age on treatment outcome. The results from coronal restoration were dichotomized to direct (composite) and indirect restoration (crowns, post and core, bridge abutments).

Chi-squared test was used for comparison of groups, ANOVA with Friedman’s test between groups and Cronbach’s alpha test for inter-operator reliability. A binary logistic regression analysis was performed to test for significance, and odds ratio and confidence interval for tooth extraction on different variables. A *p*-value ≤ 0.05 was considered statistically significant. Descriptive results are also presented.

## 3. Results

### 3.1. The Study Cohort

A total of 1149 RCTs were performed between 2012 and 2014. Of these, 462 teeth (40.2%) were self-assessed as Grade A and confirmed by instructors. Table 3 shows the distribution of the 1149 root-filled teeth by quality assessment and year. The most frequent category was Grade B where minor adverse event(s) was registered, but where the quality was otherwise satisfactory. Unsatisfactory quality (group C and D) was registered for 32 (2.7%) of all root-filled teeth.

Among the 462 teeth graded as A, 350 (75.8%) were registered with a recall appointment at least one year after treatment. The final study group consisted of 350 treated teeth.

### 3.2. Evaluation and Inter-Operator Reliability

The agreement between the two observers, including all the 318 remaining teeth, was 94% with a Kappa value of 0.625 and a significance level of <0.001. Eight disagreement cases were related to periapical lesions (uncertain cases) that were under healing but not completely resolved. After joint evaluation, agreement was reached for all cases.

### 3.3. Demographics

The age of the patients when treatment started varied from 11 to 93 years, with a mean age of 56.1 ± 15.2 years. There was no significant gender difference amongst the patients recorded in Grade A. Demographic data is presented in Table 4.

### 3.4. Outcome Failures

**Figure 1:**
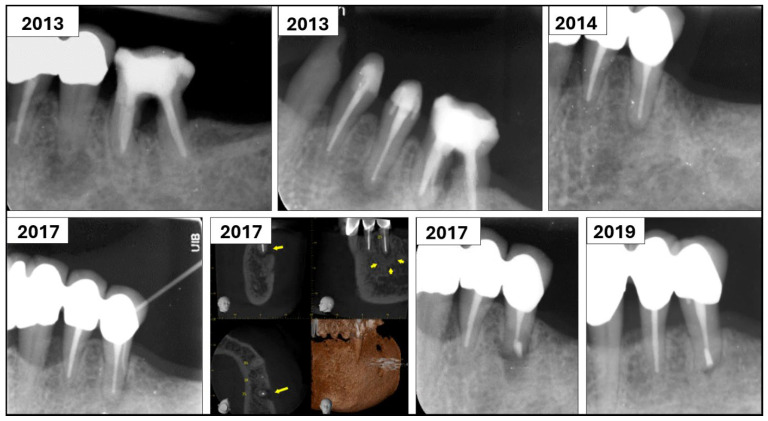
Case 3 belonged to a 67-year-old patient who was diagnosed with necrotic pulp and chronic apical periodontitis on tooth 35. Following cleaning, shaping, and intracanal dressing for two weeks, the root canal was filled with gutta-percha and AH-plus sealer. At one-year recall, there were no symptoms from the tooth, and apical radiolucency had healed. However, in 2017, the tooth was diagnosed with periapical abscess with sinus tract. A cone beam CT image showed a periapical lesion (marked with yellow arrows). Endodontic surgery was performed on the tooth at the postgraduate clinic. Recall from 2019 showed resolution of the periapical lesion and the tooth was symptom-free.

A total of 6 (1.7%) of the 350 teeth that failed, but were not extracted, were monitored for up to nine years. Five of these teeth received additional surgical retreatment (Table 5 and Figure 1). These five teeth had pulp necrosis with periapical periodontitis as preoperative diagnosis and received endodontic surgery during the follow-up period. One tooth was scheduled for observation without further treatment.

### 3.5. Extracted Teeth

**Figure 2:**
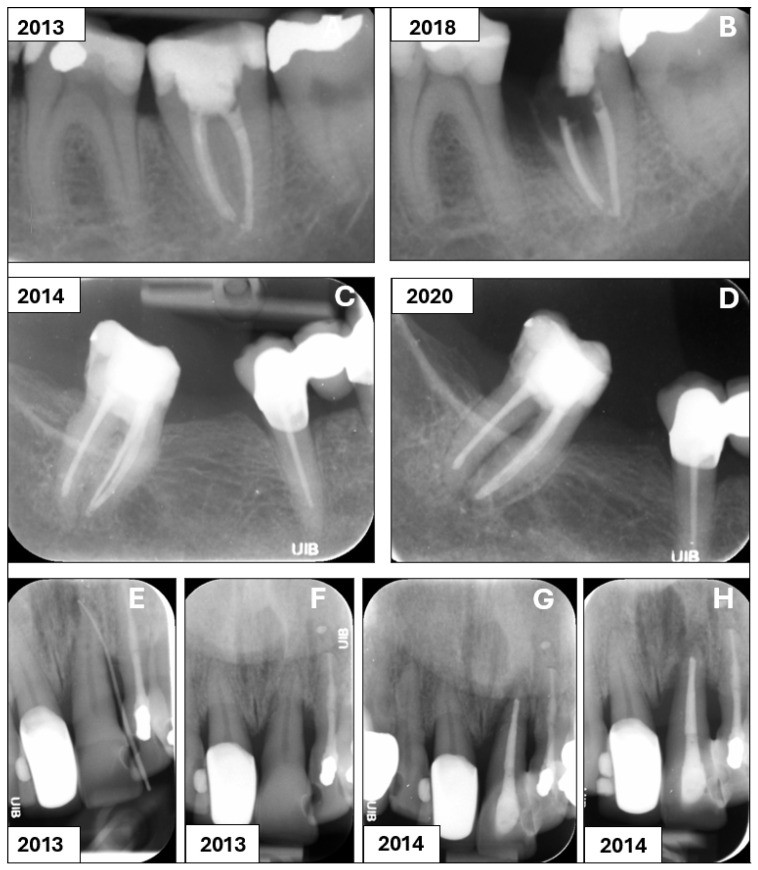
Representative radiographs of teeth extracted due to carious lesion (**A**,**B**), tooth fracture (**C**,**D**) and periodontal disease (**E**–**H**). Tooth 37 was root filled in 2013 (**A**). An expanding carious lesion resulted in an unrestorable tooth that eventually was extracted in 2018 (**B**). Tooth 47 was root filled in 2014 (**C**). In 2020, the patient experienced severe pain of sudden onset for 2 days. Clinical examination showed endodontic pocket to apex and tooth was extracted due to fracture (**D**). Tooth 21 was diagnosed with pulp necrosis and periapical abscess with sinus tract (**E**,**F**). Tooth was root filled in 2013, and a six-month (**G**) and one-year recall (**H**) showed progression of periodontal disease. Tooth 21 was eventually extracted.

All 32 extracted teeth were removed for non-endodontic reasons, including unrestorable carious lesion (38%), progression of periodontal disease (28%) or tooth fracture (34%). Representative radiographs are presented in Figure 2.

After exclusion or inclusion of the 32 teeth extracted for non-endodontic reasons, the success rate of the remaining teeth was 98.1% and the final tooth survival 90.9%, with a mean follow-up period of 2.81 years.

### 3.6. Age and Treatment Factors

In a logistic regression analysis, patients aged 56 and above had significantly (approximately 8 times) higher odds ratio of tooth extraction compared to patients under 56 years of age (*p* < 0.001). Additionally, teeth with direct restorations had 3.5 times higher odds of being extracted (*p* < 0.01) (Table 6). Significantly more teeth (77.5%) in the retreatment group received indirect coronal restoration with post and core, full coverage crown, or served as bridge abutment (*p* < 0.01).

### 3.7. Case Difficulty Assessment

**Figure 3:**
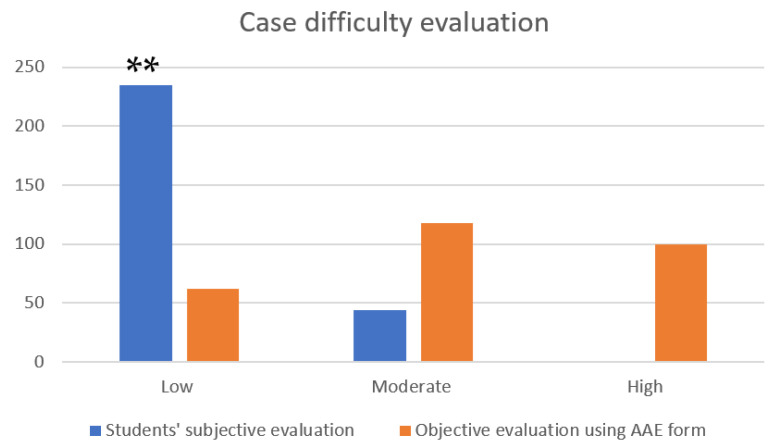
Graph showing number of teeth (*y*-axis) evaluated as low, moderate and high difficulty level (*x*-axis) by students (subjective) and using the American Association of Endodontists case difficulty evaluation form (objective). Statistical analysis using ANOVA with Friedman’s test, ** *p* < 0.01.

Out of 350 teeth, case difficulty assessment as a self-reflection exercise was performed postoperatively on 280 teeth. Unfortunately, 20% of students did not perform this self-assessment of case difficulty, although it was mandatory. Students’ subjective self-assessment was poorly correlated with an objective evaluation using the AAE form (Cronbach’s alpha = 0.32). Students evaluated 235 teeth as low, 44 as moderate and only 1 tooth at high difficulty level (Figure 3). Significantly more students evaluated the cases they had treated at low difficulty level (*p* < 0.01). Case difficulty of a tooth did not affect the treatment outcome (Table 6).

## 4. Discussion

The main findings indicate that when RCT is performed to a high standard, aimed at ensuring optimal prognosis, endodontic failure from persistent or new infection occurs in fewer than 2% of cases, yielding a success rate of 98.1% among surviving teeth. Although the goal of endodontic treatment is to preserve teeth, tooth loss occurred in approximately one in ten cases (9.1%) due to factors unrelated to endodontic infection, such as unrestorable carious lesions, periodontal disease, or structural issues related to occlusal load. Furthermore, patients under the age of 56 and teeth restored with indirect coronal restoration demonstrated significantly higher tooth survival rates. Finally, this study shows that students underestimate case difficulty of a tooth.

Quality-assessment requires a significant level of discipline, both to initiate the process and to uphold honesty throughout [2,12]. The self-assessment manual was developed with the objectives to help dentists provide better patient care, offer an up-to-date and concise foundation for general dental practice, create a structured framework for self-assessment and encourage reflection on patient care using treatment outcome as a basis for the reflection [1,2]. Grade A reflects treatments carried out without any adverse event, thus assuming that the clinical outcomes or prognosis could be predicted as optimal. Conversely, Grade D represents endodontic treatment with the lowest prognostic expectations [10]. The aim of implementing self-assessment in an academic setting is to encourage future practitioners to continuously evaluate their own performance and strive to maintain a high standard of treatment care.

Despite strictly following clinical standard of care, only 40.2% of the RCTs were graded as A. In 27 cases (2.3%), Grade C was given, meaning that an adverse outcome is anticipated. In five patients (0.4%), the treatment result was Grade D, where irreversible damage to the tooth and surrounding structures had already or would likely occur. In the years 2012–2014, case difficulty self-assessment was undertaken after treatment completion as part of self-reflection. Among all teeth assessed for case difficulty, most students (83.9%) classified the treatment as easy to perform (low difficulty level), 15.7% considered it moderately difficult and only one student (0.4%) rated a case as highly difficult. Interestingly, 22.9% of the teeth were non-surgical orthograde retreatment cases, and this, according to the AAE case difficulty assessment form, should be highly difficult to treat [11]. Furthermore, first and second molars, comprising 23% of the cases, are placed in the moderate and high difficulty category, respectively. Case difficulty assessment is an integral part of clinical decision-making. Students’ inability to accurately self-assess case difficulty is not unexpected, as clinicians too often misjudge the balance of benefits and harms in clinical decision-making, typically underestimating harms and overestimating benefits [13]. Patients similarly tend to overestimate treatment benefits [13]. Case difficulty assessment ensures that patients are informed about the challenges and risks associated with high difficulty cases, which carry a twofold risk of adverse events such as canal transportation or overfilling with gutta-percha [6]. Inaccurate perceptions of difficulty can otherwise result in suboptimal clinical decision-making, such as attempting treatment beyond one’s ability. The fact that 20% of students did not perform a self-assessment of case difficulty, combined with a tendency to classify many cases at low difficulty level, suggests a general lack of diligence in evaluation.

Case difficulty assessment before a treatment procedure provides a better opportunity to perform risk assessment, take preventive measures and approach the treatment with greater caution, ultimately aiming to reduce the number of mishaps [6,14]. Generally, endodontic cases treated in the student clinic have gradually increased in difficulty due to a greater demand for retaining teeth in the population [6,15]. Case difficulty level may be one factor explaining the decrease in the percentage of teeth graded as A. Fifteen years previously, the percentage of cases graded A was between 50 and 60%, whereas cases graded as B were around 40% [10].

Preoperative factors such as gender, ethnicity, age, and medical status have generally no influence on prognosis [16]. However, it has been shown that tooth type had a significant influence on the outcome of RCT [17]. In a systematic review from Ng et al., tooth type was shown to affect the long-term result [18]. Non-molars had a significantly higher survival rate after RCT. In addition, pulpectomies, absence of periapical periodontitis and preoperative symptoms had a significant positive effect on the long-term result. Despite previous findings, showing no impact of age on the treatment outcome [19], our study showed that age was the only preoperative factor that significantly affected treatment outcome. The negative impact of age is suggested to be associated with various comorbidities, including periodontal disease, heavily restored teeth that are more prone to fracture, and possible parafunctional habits such as bruxism, which can also lead to tooth fractures. Additionally, age-related health challenges may result in reduced adherence to oral hygiene routines due to illness or physical limitations. This study did not find any effect of tooth type, tooth location, treatment type or even case difficulty on tooth survival. No effect of case difficulty is not surprising, since case difficulty is primarily related to quality [6]. However, one postoperative factor, the choice of coronal restoration, particularly placement of an indirect coronal restoration, had a significant positive effect on tooth survival. The type of coronal restorations was evenly distributed among the patients. Interestingly, retreated teeth received significantly more indirect coronal restorations. This may be due to performance of retreatment in conjunction with prosthodontic treatment planning, which agrees with a previous study from our academic institution [20]. Furthermore, teeth with poor prognosis, such as those affected by periodontal problems, may have been restored directly rather than receiving a costly prosthodontic treatment. However, decisions regarding coronal restoration require a multidisciplinary approach, which, as indicated in this study, highlights the need for revision of current practices.

It is important to consider the difference between endodontic success and survival of a tooth. Survival in this regard is defined as the presence of a tooth after a certain time, independent of periapical findings. Successful outcome includes many factors, such as radiological and clinical findings, as well as symptoms experienced by the patients [21]. The study by Ng et al. from 2010 showed a survival rate of 93% following traditional endodontic treatment after 5 years, and 75–89% after 10 years [18]. A more recent study from 2022 showed that the 11-year cumulative survival rate for non-surgical root canal treatment was 88.37% [22]. The survival rate in this study was 90.9% after extraction of 32 teeth. The success rate was in line with another recent study performed by Almohareb et al., showing a success rate of 88% in cases with high quality, compared to 71% for cases with poorer quality of the root filling [23]. In the six teeth recorded as failures after RCT, the main reason was persisting apical periodontitis. This accounted for under 2% of the total number of teeth included in this study. Interestingly, all these cases were diagnosed with necrotic pulp and periapical periodontitis preoperatively. This underscores that endodontic infections are the primary cause of treatment failure, even when treatment is performed to a high standard. Five of these six cases received surgical retreatments after follow-up, which further improved the overall success rate. This study is also in agreement with previous findings, concluding that the most common reason for extraction of endodontically treated teeth is related to tooth structure loss due to recurrent caries, coronal fractures, and periodontal disease leading to challenges in retaining the tooth with reasonable efforts [18,24,25,26]. Furthermore, postoperative prosthodontic complications and patients’ subjective experiences are claimed as reasons for extracting endodontically treated teeth [27]. These studies, however, did not differentiate endodontic treated cases with or without adverse events.

### Strengths and Limitations

Selection bias is a relevant issue, as 350 (75.8%) out of 462 teeth graded as A were included in the study. The primary reason for attrition was patient non-attendance at scheduled follow-up visits, commonly encountered in longitudinal research. Importantly, the strength of the present study lies in its homogeneity, as all included treatments were performed to a high standard, thereby minimizing or reducing the confounding effect of treatment quality on outcome. Furthermore, it is not unexpected that asymptomatic patients may perceive no need for additional follow-up. Focusing exclusively on Grade A treatments can be viewed as both a limitation and a strength. While this approach restricts the generalizability of the findings to cases of lower quality, it ensures that the reported outcomes reflect the prognosis expected under optimal treatment conditions. Endodontic treatment in an academic clinical setting may also differ from general practice, as instructors often intervene when students encounter difficulties. This level of support may not be available in general dental practice [6]. In this context, studies have indicated that general dental practitioners may sometimes compromise on the standard of care, with deviations from the ideal standard of root canal filling [28]. According to Dahlström et al., rather than continuously striving for the optimal outcome, dentists often establish case-by-case thresholds of acceptability, providing treatment that is considered “good enough” [28]. The findings of the present study emphasize the importance of reinforcing high treatment standards early in dental education to prevent the normalization of suboptimal care in professional practice. Implementing self-assessment and preoperative case difficulty evaluation provides students with the key message to consistently strive for high-standard treatments and to avoid undertaking difficult cases beyond their current level of preparedness.

## 5. Conclusions

High-standard endodontic treatments can achieve a high success rate. However, tooth loss due to non-endodontic factors remains a challenge. Self-assessment of endodontic treatment and accurate assessment of case difficulty using relevant tools serves as an important educational aid, potentially contributing to enhanced knowledge and improved clinical decision-making.

## Figures and Tables

**Table 1:** Grading of quality according to international standards [2].

Grade A	A standard of excellence where the treatment is carried out without deviations from the ideal result. The prognosis is optimal.
Grade B	The minimum acceptable standard below which there is potential for damage to the patient resulting from one or more minor adverse event(s). The adverse event(s) may affect the prognosis.
Grade C	A treatment result with deviations that has the potential for damage, or where any damage caused is still reversible after treatment. The treatment is of unsatisfactory quality and will possibly affect the prognosis.
Grade D	A treatment result in which a patient is suffering or will possibly suffer irreversible damage in the future. The treatment is of unsatisfactory quality.

**Table 2:** Adverse events recorded and used during assessment and grading of quality.

Diagnosis related	Misdiagnosis or treatment of wrong tooth
Access related	Under- or overextension of the access cavity Canals not located Aseptic deviation such as rubber dam leakage
Instrumentation related	Root perforation—furcation, lateral or apical Transportation Instrument separation Loss of working length, ledge formation Extrusion of calcium hydroxide or sodium hypochlorite Exacerbation
Obturation related	Short root filling >2 mm from radiographic apex Overextension of gutta percha beyond radiographic apex Significant extrusion of sealer Root fracture Inadequate root filling and others

**Table 3:** Total number of root-filled teeth between 2012 and 2014, categorized according to year and grading from A to D. Parenthesis represents percentage of teeth according to year.

Year	Number of RCTs	A	B	C	D
2012	365	184 (50.4%)	169 (46.3%)	11 (3.0%)	1 (0.3%)
2013	399	146 (36.6%)	243 (60.9%)	8 (2.0%)	2 (0.5%)
2014	385	132 (34.3%)	243 (63.1%)	8 (2.1%)	2 (0.5%)
Total	1149 (100%)	462 (40.2%)	655 (57.1%)	27 (2.3%)	5 (0.4%)

**Table 4:** Demographic data on teeth self-assessed as Grade A with recall, *n* = 350. * Refer to Table 5.

Variables	Number (Percentage)
***Gender***	
Male	186 (53.1)
Female	164 (46.9)
***Diagnosis***	
Pulpitis	94 (26.9)
Necrotic pulp	177 (50.3)
Necrotic pulp with	151 (43.1)
chronic apical periodontitis	
Previously root-filled teeth	80 (22.9)
***Treatment outcome***	
Success	312 (89.2)
Failure * (not extracted)	6 (1.7)
Extracted	32 (9.1)

**Table 5:** Unsuccessful treatments according to tooth number, preoperative diagnosis, reason for failure, follow-up and further treatment.

Case	Tooth Number	Preoperative Diagnosis	Reason for Failure	Follow-Up (Years)	Further Treatment
1	34	Necrotic pulp	Periapical infection	3	Endodontic
		Chronic apical periodontitis			surgery
2	25	Necrotic pulp	Periapical infection	1.5	Endodontic
		Chronic apical periodontitis			surgery
3	35	Necrotic pulp	Periapical abscess	6	Endodontic
		Chronic apical periodontitis	with sinus tract		surgery
4	21	Necrotic pulp	Periapical abscess	8	Endodontic
		Periapical abscess with sinus tract	with sinus tract		surgery
5	23	Necrotic pulp	Periapical infection	9	Endodontic
		Chronic apical periodontitis			surgery
6	21	Necrotic pulp	Periodontitis	3.5	Observation
		Chronic apical periodontitis			

**Table 6:** Results from the logistic regression analyses for tooth extraction, with variables and frequency for gender, age, tooth type, tooth location, treatment performed, number of treatment visits, coronal restoration and case difficulty assessment using the American Association of Endodontists case difficulty form [11]. Odds ratio and 95% confidence interval (CI), NS = no significance. * represents statistical significance (*p* < 0.01).

Categories	Variables	Frequency	Odds Ratio (95% CI)	Significance
Age	Below 56 years of age	156	1	
	56 years and older	194	8.49 (3.11–23.12)	* *p* < 0.001
Tooth type	Anterior	140	1	
	Premolar	129	0.93 (0.38–2.30)	NS
	Molar	81	1.17 (0.44–3.14)	
Tooth location	Maxillary	204	1	
	Mandibular	146	0.88 (0.40–1.93)	NS
Treatment type	Pulpectomy	94	1	
	Treatment of necrotic pulp	176	1.39 (0.44–4.45)	NS
	Retreatment (orthograde)	80	1.82 (0.65–5.05)	
Number of	1	36	1	
treatment visits	2	194	2.17 (0.49–9.68)	NS
	3	86	1.28 (0.25–6.64)	
	4	21	0.85 (0.07–9.98)	
	5	13	1.42 (0.12–17.01)	
Final coronal	Indirect restoration	193	1	
restoration	Direct restoration	157	3.53 (1.60–7.78)	* *p* < 0.01
AAE case	Low	62	1	
difficulty level	Moderate	118	0.56 (0.21–1.42)	NS
	High	100	0.55 (0.18–1.39)	

## Data Availability

The raw data supporting the conclusions of this article will be made available by the authors on request.

## Supplementary Materials

The following supporting information can be downloaded at: https://www.mdpi.com/article/10.3390/dj13120571/s1, The clinical protocol used in this study.
