# Supplementary material for: High-Standard Non-Surgical Endodontic Treatment and Outcome: A Retrospective Follow-Up Study on Self-Assessment of Grading and Case Difficulty in an Academic Setting
Source: Dent J (Basel). 2025 Dec 2;13(12):571. doi: 10.3390/dj13120571 (PMC12732138; doi:10.3390/dj13120571)
Supplement: Supplementary file 1 [file dentistry-13-00571-s001.zip › dentistry-3990277-supplementary.pdf]

Treatment Protocol

All patient treatments at the undergraduate clinic of the Department of Clinical Dentistry, University of Bergen, Norway, are performed under instructor supervision at a student-to-instructor ratio of 6:1. Patients from the Patient Reception Unit are randomly assigned to fourth-year or final-year undergraduate students.

At the initial visit, a comprehensive clinical examination and digital radiographic assessment (Digora® Optime, Soredex) are conducted, followed by diagnosis, treatment planning, and obtaining informed consent. Each treatment session may last up to 150 minutes. All endodontic procedures are performed under rubber dam isolation. Students have access to an operating microscope when canal orifice location is challenging.

Working length is typically estimated using an apex locator and confirmed radiographically. Once approved, students consult with their instructor regarding the final apical size and instrumentation technique. All hand and rotary files are single-use and discarded after each session.

Root canals are irrigated with a minimum of 10 mL of Dakin's solution (0.5% buffered sodium hypochlorite), followed by 5 mL of 17% EDTA to remove the smear layer prior to intracanal medication or obturation. In retreatment cases with apical periodontitis, Lugol's solution (5% iodine/potassium iodide) is applied for 10 minutes before final irrigation and obturation.

Treatment sessions conclude with placement of intracanal calcium hydroxide medicament (Ultracal™ XS™, Ultradent Products, Inc., USA) and an intermediate restorative material (IRM® Caps™, Dentsply DeTrey GmbH, Konstanz, Germany). A minimum two-week period is recommended for medicament placement. Radiographic documentation is mandatory, with at least five periapical radiographs taken: preoperative, working length, master cone, condensation, and final.

Final obturation is performed using the cold lateral condensation technique with a standardized gutta-percha master cone, accessory points, and AH Plus® sealer (Dentsply DeTrey GmbH, Konstanz, Germany). Patients are subsequently referred to the restorative or prosthodontic clinic for post-endodontic restoration.

Following completion of treatment, all endodontically treated teeth are assessed during a clinical evaluation session involving the student and supervising instructor. For each treated tooth, the student completes a self-evaluation form, which is subsequently reviewed and discussed with the instructor prior to accreditation.

The self-evaluation aims to document case difficulty, record any procedural mishaps, grade the treatment according to the SAMS (Self-Assessment Manual and Standards for Health Services) protocol, and predict prognosis based on the treatment provided. After this appraisal, patients are scheduled for a one-year recall appointment.
